# Changes in physical activity outcomes in the Strong Hearts, Healthy Communities (SHHC-2.0) community-based randomized trial

**DOI:** 10.1186/s12966-022-01401-1

**Published:** 2022-12-28

**Authors:** Jay E. Maddock, Margaret Demment, Meredith Graham, Sara Folta, David Strogatz, Miriam Nelson, Seong-Yeon Ha, Galen D. Eldridge, Rebecca A. Seguin-Fowler

**Affiliations:** 1 School of Public Health, Texas A&M University, College Station, TX 77843 USA; 2 Texas A&M AgriLife Research and Extension Center, Dallas, TX 75252 USA; 3 Friedman School of Nutrition Science and Policy, Tufts University, Boston, MA 02155 USA; 4 Bassett Healthcare Network, Cooperstown, NY 13326 USA; 5 Newman’s Own Foundation, Westport, CT 06880 USA; 6 Department of Statistics, Texas A&M University, College Station, TX 77843 USA; 7 Institute for Advancing Health Through Agriculture, Texas A&M AgriLife, College Station, TX 77843 USA

**Keywords:** Physical activity, Rural, Community-based, Randomized controlled trial

## Abstract

**Background:**

Physical inactivity is a risk factor for numerous adverse health conditions and outcomes, including all-cause mortality. Aging rural women are at particular risk for physical inactivity based on environmental, sociocultural, and psychosocial factors. This study reports on changes in physical activity and associated factors from a multicomponent community-engaged intervention trial*.*

**Methods:**

*Strong Hearts, Healthy Communities 2.0* (SHHC-2.0) was a 24-week cluster (community) randomized controlled trial building on the results from the previous trial of SHHC-1.0. Rural women (*n* = 182) aged 40 and over living in 11 rural communities in upstate New York were recruited. The intervention consisted of twice-weekly experiential classes focused on exercise, nutrition, and civic engagement. Physical activity outcomes included accelerometry and self-report as well as related psychosocial measures at midpoint (12 weeks) and post-intervention (24 weeks). Data were analyzed using multilevel linear regression models with the community as the random effect.

**Results:**

Compared to participants from the control communities, participants in the intervention communities showed a significant increase in objectively measured moderate to vigorous intensity physical activity: at 12 weeks (increase of 8.1 min per day, *P* < 0.001) and at 24 weeks (increase of 6.4 min per day; *P* = 0.011). Self-reported total MET minutes per week also increased: at 12 weeks (increase of 725.8, *P* = 0.003) and 24 weeks (increase of 955.9, *P* = 0.002). Several of the psychosocial variables also showed significant positive changes.

**Conclusions:**

The SHHC-2.0 intervention successfully increased physical activity level and related outcome measures. Modifications made based upon in-depth process evaluation from SHHC-1.0 appear to have been effective in increasing physical activity in this at-risk population.

**Trial registration:**

Clinicaltrials.gov: NCT03059472. Registered 23 February 2017.

**Supplementary Information:**

The online version contains supplementary material available at 10.1186/s12966-022-01401-1.

## Background

There is vast literature demonstrating a dose–response relationship between physical activity and improved health and well-being [1]. While physical activity is vital across gender and age groups, women and older adults tend to get less physical activity than men and younger adults [2]. The Physical Activity Guidelines for Americans recommend that adults, including older adults, get 150 min per week of moderate intensity physical activity or 75 min of vigorous intensity physical activity and perform muscle-strengthening activities on at least two days a week [3]. Less than 20% of American women meet these guidelines, with older women [3, 4] and women living in rural areas even less likely to meet them [5, 6].

Rural communities have the most robust relationship between the recreation environment (including access to exercise facilities, parks, and schools) and meeting physical activity guidelines compared to more urban counties [7]. However, even if physical activity facilities are available, middle age and older women may be less inclined to use co-ed exercise facilities [8–10]. The social environment and support offered by sex- and age-specific groups is an essential facilitator of adoption and maintenance of physical activity among women [11–13].

Social and cultural norms and attitudes further challenge being physically active in rural areas [8, 14, 15]. Active transport, riding a bicycle or walking, is seen as potentially stigmatizing and negative, e.g., may be associated with a loss of driver’s license [16]. Family obligations are a common barrier to being physically active for women who frequently assume traditional caregiving roles and prioritize time with their kids and husbands over exercise [8]. In contrast, social interaction appears to be an important facilitator of active lifestyles in rural areas, particularly for women; organized group activities, such as walking, are viewed as an opportunity to socialize with friends and connect with the community [8].

Effective, evidence-based interventions are needed for rural, middle-aged and older women that address cardiovascular and muscle strengthening physical activity guidelines and the social and environmental barriers and facilitators to meeting them. The National Cancer Institute only lists two evidence -based programs for rural women: one specifically designed for Black women and the other StrongPeople Living Well (formerly known as StrongWomen—Healthy Hearts), a predecessor to the program in the present study [17].

Between 2015 and 2016, a randomized community trial called *Strong Hearts, Healthy Communities* (SHHC-1.0) was conducted with women living in 16 rural communities in Montana and New York [18]. The multicomponent, multi-level intervention consisted of twice-weekly exercise and nutrition classes across 24 weeks. The socioecological model was used to target behavior change at the individual, social, and community levels. For example, the curriculum included lessons to support participants in developing knowledge, skill mastery, and autonomy related to physical activity. There were out-of-class materials to help participants self-monitor adherence to the program and to engage friends and family in their new activities to encourage social support for behavior maintenance. Classes included aerobic and strength-based exercise and a civic engagement component to address built environment issues in the community. Intervention participants from SHHC-1.0 demonstrated significant positive changes in BMI (kg/m^2^), C-reactive protein, and Life’s Simple 7, a cardiovascular risk score; they also significantly improved in measured functional fitness outcomes such as increased strength and endurance [19, 20]. However, physical activity impacts were minimal: data indicated a significant increase in walking for the intervention group, but no overall increase in self-reported physical activity possibly due to an increase in variability in vigorous intensity physical activity; there were no significant difference between groups on accelerometry-measured outcomes [19].

A process evaluation of SHHC-1.0 showed that the program had been delivered with a high level of fidelity and that, on average, participants attended 2/3 of the classes for about 38 contact hours [21]. Qualitative analyses indicated that many women had trouble keeping up with fast-paced cardiovascular dance routines and preferred slower-paced walking alternatives [21]. Recommendations included more standardization of the exercise programs, a greater variety of exercise videos, and exercise modifications for participants with mobility issues.

Given the overall positive outcomes from the SHHC-1.0 trial, the investigative team reviewed the process data and made substantive changes to improve the behavioral targets within the program. These changes included: the inclusion of a greater variety of aerobic exercise DVDs, including at-home and online options; more consistency for strength training in class by ensuring that exercises were varied and repeated equally throughout the program; the addition of a Health Journal for participants to record exercise goals and progress; additional curriculum content in the Participant Guide, including instructions for each strength training exercise and additional handouts developed for each class; out of class strategies/encouragement for participants to engage in program-related activities (particularly strength training and aerobic exercise) during non-class time; and additional content related to social support and sabotage for increasing physical activity, as well as dealing with difficulty in this area.

This resulted in the SHHC-2.0 program and second randomized trial designed to assess the effects of the modified intervention on multiple, modifiable cardiovascular disease risk factors and outcomes among rural women.

### Objectives

The objective of the analysis reported herein was to examine the effects of the SHHC-2.0 intervention program on physical activity outcomes between intervention and control groups. Specifically, the objectives were:

1. Assess changes in objective (i.e., accelerometer) and self-reported physical activity between the intervention and control groups at 12 weeks and 24 weeks.
2. Assess changes in self-efficacy, social support, and attitudes towards physical activity between the intervention and control groups at 12 weeks and 24 weeks.
3. Both of these objectives were also completed in a subsample of women 60 years of age or older to assess the effectiveness of the intervention in an older sample.

## Methods

### Study design

This study employed a two-group randomized design in rural upstate New York communities. Medically underserved communities with a Rural–Urban Commuting Area code (RUCA) of 4 or higher (micropolitan or rural) were selected. Community sites were pair-matched based upon population size (394 to 8,836) and RUCA code (4.1 to 10.2) [22]. Communities were geographically distinct and were not involved in the original SHHC trial (SHHC-1.0).

Following baseline assessments, communities were randomly assigned in pairs to intervention (*n* = 6) and control (*n* = 5; delayed intervention) groups by a statistician who was not involved further in the study. Complete study details have been published elsewhere and are only briefly recapped here [23, 24].

### Intervention

The original SHHC-1.0 intervention was created based on extensive community input, including focus groups, surveys, and community audits. Results from the original trial and the process evaluation led to the creation of the refined SHHC-2.0 intervention [21, 25]. The SHHC-2.0 program consists of twice-a-week 60-min experiential group physical activity and nutrition education classes along with sessions on social and environmental change over 24 weeks; the intervention programming started in Spring/Summer of 2017 (April – June) and finished in Fall 2017 (September –November) [23].

Cooperative Extension health educators (herein Extension educators) led classes at various community locations, including libraries, town halls, churches, and other public buildings. The study team provided exercise equipment (low-weight dumbbells, yoga mats), aerobic exercise videos, a Leader Toolkit, Participant Guides, and Health Journals (for participants). Extension educators attended an intensive one-day training and had weekly support calls during active program implementation. Fidelity was assessed by leader questionnaires completed after each class and community site visits conducted by trained staff.

#### Physical activity specific intervention description

The general goals for the physical activity portion of the curriculum were based on guidelines from the Office of Disease Prevention and Health Promotion [26]: (1) Minimize sedentary time; (2) Strength train at least two days per week; (3) Engage in moderate to vigorous intensity exercise at least five days per week; and (4) Be as physically active as possible daily (taking the stairs, walking farther in a parking lot, etc.).

Forty-five of the 48 classes included progressive strength training and aerobic exercise, which averaged 13 min of strength training per class and 24 min of aerobic exercise per class. Aerobic exercise included walking and aerobic dance DVDs, which progressed from low intensity to moderate intensity ranging from 15 to 30 min per class (e.g., walking slowly to walking intervals with brisk walking or light jogging). The progressive strength training program included warm-up and cool-down stretches (four exercises) ranging from 10 to 20 min per class. Exercises were done in two sets of ten repetitions for a slow 8-s count. The topics covered in class progress over time from introductory strength training and aerobic exercise concepts to sustaining physical activity strategies. The beginning of the program is focused on behavior change, then the focus turns to examine the difficulties and accomplishments of behavior change, and finally how to sustain behavior change.

Participants were also encouraged to engage in physical activity outside of class time, including low to moderate intensity exercise, moderate to vigorous intensity exercise, strength training, and being as physically active as possible. The curriculum provided strategies and recommendations for exercising in bad weather and included programming for both indoor and outdoor options.

### Study sample

Enrollment occurred between January and June of 2017. Extension educators recruited participants using flyers, radio advertisements, newspaper articles, social media, and word of mouth. Inclusion criteria included being female, aged 40 years and older, and either: (1) overweight (BMI = 25–30) and sedentary (no more than one bout of > 30 min of leisure physical activity per week on average, during the past three months) or (2) obese (BMI > 30). Women were excluded if they did not provide informed consent or permission from a healthcare provider, had systolic blood pressure > 160 mmHg or diastolic blood pressure > 100 mmHg, had a resting heart rate < 60 or > 100 bpm, had a cognitive impairment, as determined by a 6-item cognitive screening test [27], were participating or planning to participate in another health behavior change program in the next 12 months or were unwilling to be randomized to either group. Once the paired sites had enough participants with baseline data collection complete, then those sites were randomized and intervention site could begin. The Cornell University and Bassett Healthcare Network Institutional Review Boards approved all study procedures.

In accordance with the design of the first trial [18], this trial was powered at 80% to detect the primary outcome, mean change in weight from baseline to 24 weeks between groups of 1.95 kg, with a two-sided alpha, accounting for intraclass correlation of clusters and 15% attrition. It was not specifically powered for the analyses in this report.

### Measures

Participants were asked to wear an accelerometer and complete an online survey via Qualtrics on demographics, physical activity behaviors, and psychosocial physical activity measures just prior to the start of the intervention (baseline), at 12 weeks (halfway through the program), and immediately following the 24-week program (post-intervention).

The measures are detailed below:

- Light intensity physical activity and moderate to vigorous intensity physical activity (MVPA) were measured as continuous variables (averaged to minutes per day) using the accelerometer ActiGraph Model GT3XE (ActiGraph LLC, Pensacola, FL). Participants were instructed to wear the device at the hip for seven days and only to remove it when sleeping, bathing, or swimming. Data were recorded at 30 Hz and analyzed using an epoch length of 60 s. The low-frequency extension filter was used, and non-wear time was excluded by Choi et al. algorithm [28]. Daily level data were excluded if wear time was less than 10 h (600 min) in a day. Freedson cut points were used for physical activity intensity [29].
- A dichotomous variable was then created to test if participants met aerobic physical activity recommendations of greater than or equal to 150 min of MVPA per week.

In addition to the accelerometer measures, we collected self-report physical activity data to include such activities as swimming, where the accelerometer could not be worn, or weightlifting, which is strenuous but generates few steps. The International Physical Activity Questionnaire Short Form (IPAQ-SF) was used to self-report on physical activity and scored according to IPAQ’s Guidelines for Data Processing. Metabolic equivalent (MET) minutes per week were compiled according to the IPAQ analysis guidelines [30]. Sedentary behavior was self-reported using the Sedentary Behavior Questionnaire (SBQ) [31]. Self-efficacy for physical activity (Self-efficacy for sticking to exercise habits [scale 1–5]) and for making time for exercise (scale 1–5) [32], social support for physical activity (family participation in exercise [scale 10–50], friend participation in exercise [scale 10–50], and family rewards and punishment for exercise [scale 3–15]) [33] and attitudes toward exercise (combined attitude toward exercise score [scale 1–14]) were also assessed [34].

Basic demographic information was collected at baseline. Demographic questions were derived from national surveys (e.g., U.S. Census). Study participants were instructed to report any adverse events to leaders and/or the research team at any time. Survey questions about adverse events were included at 12 and 24 weeks.

### Statistical analyses

For the primary analysis, the differences in change from 0–12 weeks and 0–24 weeks between groups were analyzed using intent-to-treat linear mixed-effects multilevel models, which included random cluster (community) effects to account for the community-level randomization and correlation between participants in the same community as well as a priori covariates age and education. Two secondary analyses were done: (1) with participants 60 years and older and (2) mixed logistic regression with random cluster (community) effects tied to achieving the goal of 150 min per week of MVPA. Participants were categorized by meeting or not meeting that recommendation at the end of the intervention.

Data were missing in the following proportions at each time period for the survey: 5% at baseline, 29% at 12 weeks, and 37% at 24 weeks. For accelerometry, data were missing for the following: 3% at baseline, 31% at 12 weeks, and 35% at 24 weeks. To explore the potential that data may not be missing at random, baseline characteristics of respondents and non-respondents were compared at 24 weeks for each data collection tool (survey and accelerometer). No significant differences in accelerometer data for respondents versus non-respondents were observed for age, income, education, race, BMI, weight, meeting physical activity guidelines, or self-reported perceived overall health. For the survey, non-respondents tended to have a higher BMI compared to respondents, and therefore BMI was used as an auxiliary variable for multiple imputation (MI).

MI was used to estimate missing data and standard errors for the intention-to-treat analyses. Imputations followed standardized, rigorous procedures, including auxiliary variables (random assignment group, community site, age, education, and BMI) and employed hierarchical approaches. Fraction of Missing Information (FMI) was used to measure the level of uncertainty about the values imputed for missing values (median FMI for outcomes was 0.21 [range: 0.03 to 0.57]). We used 70 imputations, which satisfies the recommendations to have the number of imputations (at least) equal to the highest FMI percentage.

#### Sensitivity analyses

Sensitivity, “tipping point,” analyses were conducted to identify the point at which adjusting imputed values reversed the main findings and determine the plausibility of erroneous conclusions based on data not missing at random and MI modeling [35]. To determine the sensitivity of the analysis to outliers, we ran the same analyses with outliers (1.5 interquartile range above the third quartile or below the first quartile) removed. Complete case analysis was also done using restricted maximum likelihood to incorporate all available data.

Analyses were conducted in 2022 using SAS, version 9.4. The Principal Investigator had full access to all study data and takes responsibility for its integrity and the data analysis.

## Results

**Fig. 1:**
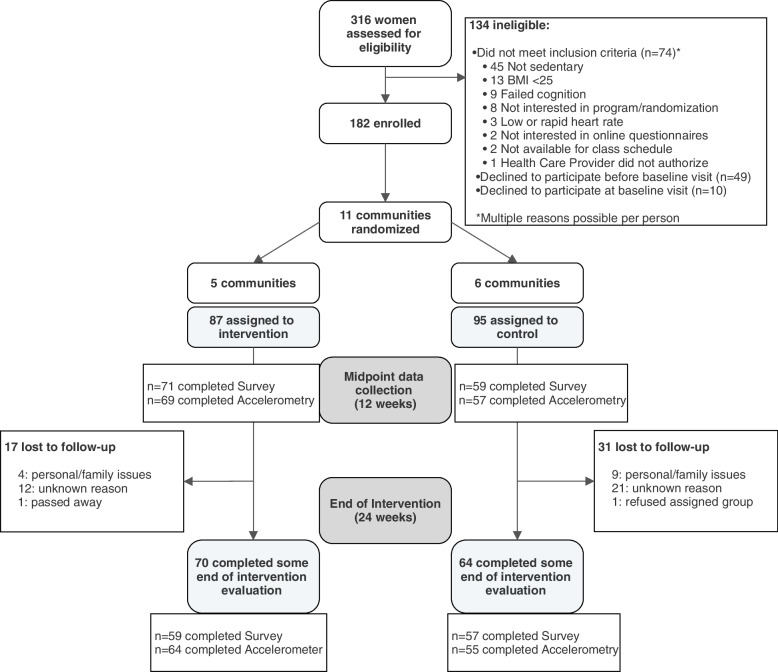
Flow chart for *Strong Hearts, Healthy Communities-2.0* randomized trial

A total of 316 individuals were screened, and 182 women (*n* = 87 in five intervention communities; *n* = 95 in six control communities) were enrolled in the trial, including 70 women aged 60 or older (*n* = 35 in intervention communities and *n* = 35 in control communities). Figure 1 displays the study flow chart. Table 1 presents baseline characteristics by group. Sixty-nine percent of intervention participants attended ≥ 50% of classes, and 38% of participants attended ≥ 75% of classes.

**Table 1:** *Strong Hearts, Healthy Communities-2.0* participant characteristics at baseline

	**Total**	**Control**	**Intervention**
**Participants, n (%)**	182 (100)	95 (52.2)	87 (47.8)
**Age, y ± SD**	57.2 ± 9.0	55.9 ± 8.5	58.5 ± 9.3
**Race/ethnicity (*n* = 168), n (%)**			
White, non-Hispanic	164 (97.6)	84 (97.7)	80 (97.6)
Non-white or Hispanic	4 (2.4)	2 (2.3)	2 (2.4)
**Income (*n* = 162), n (%)**			
< $25,000	29 (17.9)	17 (20.0)	12 (15.6)
$25,000–50,000	37 (22.8)	15 (17.6)	22 (28.6)
> $50,000	96 (59.3)	53 (62.4)	43 (55.8)
**Relationship status (*n* = 171), n (%)**			
In a relationship	116 (67.8)	62 (71.3)	54 (64.3)
Not in a relationship	55 (32.2)	25 (28.7)	30 (35.7)
**Education (*n* = 172), n (%)**			
High school or less	26 (15.1)	14 (16.1)	12 (14.1)
Some college/technical or vocational school	35 (20.3)	17 (19.5)	18 (21.2)
College graduate	63 (36.6)	33 (37.9)	30 (35.3)
Postgrad/professional	48 (27.9)	23 (26.4)	25 (29.4)
**Smoking (*n* = 171), n (%)**			
Never	100 (58.5)	49 (56.3)	51 (60.7)
Former	69 (40.4)	37 (42.5)	32 (38.1)
Current	2 (1.2)	1 (1.2)	1 (1.2)
**Overall health (*n* = 175), n (%)**			
Excellent/very good	46 (26.3)	20 (22.5)	26 (30.3)
Good	99 (56.6)	50 (56.2)	49 (57.0)
Fair/poor	30 (17.1)	19 (21.3)	11 (12.7)
**Self-report condition/disease (*n* = 170), n (%)**			
High blood cholesterol	71 (41.8)	33 (38.4)	38 (45.2)
Hypertension	71 (41.8)	41 (47.7)	30 (35.7)
Arthritis	70 (41.2)	39 (44.8)	31 (37.3)
High blood sugar	37 (21.8)	16 (19.3)	21 (24.1)
Diabetes	25 (14.7)	17 (19.5)	8 (9.6)
Cancer	12 (7.1)	5 (5.7)	7 (8.4)
Heart disease	10 (5.9)	5 (5.7)	5 (6.0)
Kidney disease	3 (1.8)	1 (1.1)	2 (2.4)
**Behaviors (Accelerometry) (*n* = 176), mean ± SD**			
Average Light Physical Activity (minutes/day)	297.9 ± 80.3	290.7 ± 71.3	305.5 ± 88.6
Average Moderate to Vigorous Physical Activity (minutes/day)	16.7 ± 14.8	17.6 ± 15.5	15.7 ± 14
**Behaviors (Survey) (*n* = 175), mean ± SD**			
IPAQ total MET minutes per week	1002.9 ± 1371.4	950.8 ± 1139.6	1056.9 ± 1581.1
IPAQ walking MET minutes per week	426.1 ± 619.5	381.7 ± 575.1	472 ± 662.5
IPAQ moderate MET minutes per week	216.6 ± 504.3	203.2 ± 361.9	230.5 ± 620.2
IPAQ vigorous MET minutes per week	360.2 ± 738	365.8 ± 607.6	354.4 ± 856
SBQ: Total sitting hours per week	63.1 ± 25.9	63.1 ± 27.5	63 ± 24.3
**Psychosocial measures (Survey) (*n* = 154–173)**^a^**, mean ± SD**			
Self-efficacy for sticking to exercise habits (scale 1 to 5)	2.9 ± 0.9	3 ± 0.8	2.8 ± 0.9
Self-efficacy for making time for exercise (scale 1 to 5)	3.2 ± 0.9	3.4 ± 0.9	3.1 ± 0.9
Family participation in exercise (scale 10 to 50)	18.2 ± 8.1	18.8 ± 8.3	17.4 ± 7.9
Friend participation in exercise (scale 10 to 50)	18.3 ± 8.2	19.8 ± 8.5	16.7 ± 7.6
Family rewards and punishment for exercise (scale 3 to 15)	3.7 ± 1.2	3.7 ± 1.2	3.8 ± 1.2
Combined attitude toward exercise score (scale 1 to 14)	10.4 ± 0.3	10.4 ± 0.3	10.4 ± 0.3

^a ^The number of responses was lower (*n* = 154–157) for the Sallis Social Support Scale for Physical Activity questions because those living alone were asked to skip the ‘family’ columns, this also led to additional missed responses because people skipped the ‘friends’ questions as well

*IPAQ* International Physical Activity Questionnaire, *MET* Metabolic Equivalent of Task, *SBQ* Sedentary Behavior Questionnaire, *SD* Standard Deviation

### Physical activity analyses

Table 2 displays changes from baseline to the midpoint (12 weeks) and end of intervention (24 weeks). The results from the complete case and MI were similar; MI estimates are described herein. (See Supplemental Tables 1 and 2 for complete case results).

**Table 2:** Within-group change and between-group change in physical activity behaviors and psychosocial measures from baseline to midpoint (12 weeks) and intervention end point (24 weeks)

	**0 to 12 weeks**				**0 to 24 weeks**			
	**Control participants** **(*n* = 95)**	**Intervention participants** **(*n* = 87)**	**Difference between groups**		**Control participants** **(*n* = 95)**	**Intervention participants** **(*n* = 87)**	**Difference between groups**	
	Mean change ± S.D	Mean change ± SD	Estimate (95%CI)	*P* value	Mean change ± S.D	Mean change ± S.D	Estimate (95%CI)	*P* value
**Behaviors (Accelerometry)**								
Average Light Physical Activity (minutes/day)	0.7 ± 8.4	26.4 ± 8.5	**25.67 (2.36,48.97)**	**0.031**	-0.4 ± 8.4	2 ± 8.4	1.21 (-21.80,24.21)	0.918
Average Moderate to Vigorous Physical Activity (minutes/day)	-0.2 ± 1.5	7.9 ± 1.6	**8.13 (3.87,12.40)**	** < 0.001**	-0.6 ± 1.7	5.8 ± 1.6	**6.43 (1.48,11.38)**	**0.011**
**Behaviors (Survey)**								
IPAQ total MET minutes per week	169.3 ± 147.8	895.1 ± 207.2	**725.77 (242.96,1208.59)**	**0.003**	93.4 ± 172	1062.4 ± 271.4	**955.90 (355.93,1555.86)**	**0.002**
IPAQ walking MET minutes per week	-23.1 ± 68.7	58.7 ± 89	81.77 (-133.59,297.13)	0.457	-24.2 ± 83.3	164.8 ± 89.3	191.50 (-47.31,430.31)	0.116
IPAQ moderate MET minutes per week	-5.5 ± 50.4	275.4 ± 77.1	**280.91 (103.36,458.46)**	**0.002**	54.3 ± 67.6	328.1 ± 111.8	**269.82 (27.01,512.63)**	**0.029**
IPAQ vigorous MET minutes per week	197.9 ± 109.6	560.9 ± 119.9	**363.09 (45.96,680.21)**	**0.025**	63.3 ± 96.2	569.5 ± 154.1	**494.58 (158.12,831.04)**	**0.004**
SBQ: Total sitting hours per week	-8.5 ± 3.3	-11.7 ± 2.8	-3.20 (-11.22,4.83)	0.434	-9.9 ± 3.5	-8.2 ± 3.5	1.67 (-7.58,10.93)	0.723
**Psychosocial measures (Survey)**								
Self-efficacy for sticking to exercise habits (scale 1 to 5)	-0.5 ± 0.1	0.1 ± 0.1	**0.54 (0.22,0.87)**	**0.001**	-0.3 ± 0.1	-0.02 ± 0.1	0.33 (-0.09,0.75)	0.124
Self-efficacy for making time for exercise (scale 1 to 5)	-0.4 ± 0.1	-0.2 ± 0.1	0.28 (-0.05,0.61)	0.091	-0.5 ± 0.1	-0.2 ± 0.1	0.29 (-0.13,0.72)	0.174
Family participation in exercise (scale 10 to 50)	-0.6 ± 1.2	3.3 ± 1.1	**3.87 (0.53,7.2)**	**0.023**	-1.1 ± 1.3	4.9 ± 1.5	**5.69 (1.78,9.61)**	**0.005**
Friend participation in exercise (scale 10 to 50)	-0.5 ± 1.5	6.7 ± 1.5	**7.18 (3,11.36)**	**0.001**	1.7 ± 1.8	6.1 ± 1.5	4.46 (-0.08,9.01)	0.054
Family rewards and punishment for exercise (scale 3 to 15)	0.04 ± 0.4	-0.1 ± 0.3	-0.11 (-1.14,0.92)	0.838	0.2 ± 0.3	0.3 ± 0.4	-0.06 (-2.18,2.06)	0.954
Combined attitude toward exercise score (scale 1 to 14)	-0.1 ± 0.05	-0.2 ± 0.04	-0.11 (-0.24,0.02)	0.094	-0.1 ± 0.1	-0.2 ± 0.1	**-0.18 (-0.34,-0.01)**	**0.033**

All estimates from MI models and adjusted for random cluster (community) effects, random assignment group, age, and education

*CI* Confidence Interval, *IPAQ* International Physical Activity Questionnaire, *MET* Metabolic Equivalent of Task, *SBQ* Sedentary Behavior Questionnaire, *SD* Standard Deviation

Bold indicates *P* value < 0.05

Intervention participants improved compared to controls in average MVPA measured via accelerometer at 12 weeks (increase of 8.1 min per day, 95%CI: 3.9 to 12.4, *P* < 0.001) and 24 weeks (increase of 6.4 min per day, 95%CI: 1.5 to 11.4, *P* = 0.011). Additionally, at 12 weeks intervention participants improved compared to controls in average light intensity physical activity (an increase of 25.7 min per day, 95%CI: 2.4 to 49.0, *P* = 0.031).

On the IPAQ, intervention participants improved compared to controls in the following self-reported behaviors at 12 weeks and 24 weeks: total MET minutes per week at 12 weeks (increase of 725.8, 95%CI: 243.0 to 1208.6, *P* = 0.003) and 24 weeks (increase of 955.9, 95%CI: 355.9 to 1555.9, *P* = 0.002). Both moderate MET minutes per week at 12 weeks (increase of 280.9, 95%CI: 103.4 to 458.5, *P* = 0.002) and 24 weeks (increase of 269.8, 95%CI: 27.0 to 512.6, *P* = 0.029) were significantly higher in intervention participants compared to controls. Vigorous MET minutes per week at 12 weeks (increase of 363.1, 95%CI: 46.0 to 680.2, *P* = 0.025) and 24 weeks (increase of 494.6, 95%CI: 158.1 to 831.0, *P* = 0.004) were significantly higher in intervention participants compared to controls.

### Psychosocial outcomes

Intervention participants significantly improved compared to controls for family participation in exercise at 12 weeks (increase of 3.9 on a 10–50 scale, 95%CI: 0.5 to 7.2, *P* = 0.023) and 24 weeks (increase of 5.7, 95%CI: 1.8 to 9.6, *P* = 0.005). Additionally, at 12 weeks, intervention participants improved compared to controls in self-efficacy for sticking to exercise habits (increase of 0.54 on a 1–5 scale, 95%CI: 0.22 to 0.87, *P* = 0.001) and friend participation in exercise (increase of 7.18 on 10–50 scale, 95%CI: 3.00 to 11.36, *P* = 0.001). Finally, an unintended finding was that combined attitudes towards exercise score decreased at 24 weeks (*decrease* of 0.18 on a 1–14 scale, 95%CI: -0.34 to -0.01, *P* = 0.033).

Findings that were significant remained so at 12 or 24 weeks when outliers (1.5 interquartile range above the third quartile or below the first quartile) were removed. (See Supplemental Tables 1 and 2 for outlier results.)

### Secondary analyses

#### 60 + subsample

In the subsample of participants 60 years and older, intervention participants improved compared to controls in average MVPA measured via accelerometer at 12 weeks (increase of 8.2 min per day, 95%CI: 2.2 to 14.1 *P* = 0.008) and 24 weeks (increase of 7.0 min, 95%CI: 0.7 to 13.4, *P* = 0.031).

As in the total sample, we also saw an increase in self-efficacy for sticking to exercise habits at 12 weeks (increase of 0.83, 95%CI: 0.31 to 1.36, P = 0.002). These results are displayed in Supplemental Table 3.

**Table 3:** Within-group percentages and between-group estimated odds ratios from mixed logistic regression of meeting physical activity recommendations at end of intervention (24 weeks)

		**Control** **(*n* = 95)**		**Intervention** **(*n* = 87)**		**Mixed Logistic Regression**^**a**^	
**Behavioral aim**	**Measure Per Day**	0 weeks # (%)	24 weeks # (%)	0 weeks # (%)	24 weeks # (%)	Odds Ratio (95%CI)	*P* value
150 min of moderate-to-vigorous physical activity per week	Average Moderate to Vigorous Physical Activity (minutes/day) greater than 21.43 min	25 (26.3)	18 (18.9)	19 (21.8)	26 (29.9)	1.99 (0.92, 4.29)	0.074

*CI* Confidence Interval, *MI* Multiple Imputation

Bold indicates *P* value < 0.05

^a^All odds ratios from MI models and adjusted for random cluster (community) effects, age, and education and estimate the odds of meeting recommendations for intervention group compared to control group

#### Behavioral aim

Less than a quarter (24.2%) of participants met the aerobic physical activity guidelines of 150 min of MVPA at baseline. Control arm participants decreased from 26.3% meeting the guidelines to 18.9% meeting the guidelines at the end of the program as measured by accelerometer. Intervention arm participants increased in meeting the recommendation from 21.8% initially meeting the guidelines to 29.9% at the end of the program. Though intervention arm participants were somewhat more likely to meet the aerobic guidelines than control arm participants, the estimate for the effect was not significant (odds ratio: 1.99, 95%CI: 0.92 to 4.29, *P* = 0.08 (Table 3). For sedentary behavior, there were no improvements between arms (Table 2).

#### Adverse events

No adverse events were reported in either group.

## Discussion

Participants in the intervention group had significantly more objectively measured and self-reported physical activity at 12 and 24-week assessments than in the control group. This finding was consistent among the subsample of 70 participants who were age 60 and older at baseline. In the original SHHC-1.0 trial, there were no significant differences in accelerometer measures between groups and only a self-reported difference in MET walking minutes per week [19]. In the current trial, there were significant differences as measured by accelerometer as well as self-reported increases in total MET minutes and moderate and vigorous MET minutes per week. These effects were clinically significant with a between-group increase of 6–8 min per day of MVPA and an 8% increase in participants meeting the aerobic physical activity guidelines. While measuring the exact effect on the participants’ health is difficult, there is a clear dose–response relationship between physical activity and cardiovascular disease [36], diabetes [37], depression and anxiety [38], and all-cause mortality [39].

An important element of SHHC-2.0 was engagement of participants’ close friends and family members—their “social network”—in the intervention, through printed and web-based materials that focus on “partnered” activities as well as the community-wide events (e.g., bike safety class). Positive changes in physical activity among participants were expected to influence attitudes, knowledge, and behavior among their social network [40, 41] and in turn keep participants motivated to sustain their physical activity changes. SHHC-2.0 intervention participants demonstrated positive improvements in family participation in exercise at both 12 and 24 weeks. This is particularly notable given that rural women often see family obligations as a barrier and have “concerns over abandoning family responsibilities to fulfill their own needs” [42]. Improvements were seen in self-efficacy for sticking to exercise habits and friend participation in exercise were seen at 12 weeks but not maintained at 24 weeks, suggesting that further iterations should find additional tools and support to maintain these positive outcomes. Physical activity environment and life stress are predictors of maintenance and relapse for physical activity. Adding materials that address staying active in poor weather and during stressful times may assist with maintenance [43].

The study did have one unexpected finding. As noted, the intervention group decreased in exercise attitudes more than the control group. The American Association of Retired People exercise attitudes questionnaire [34] includes questions such as: “I should exercise more than I do.” and “I often feel guilty about my lack of exercise.” Intervention participants may have had higher expectations for themselves in terms of exercise by the end of the program than they did before the program.

There were several limitations in this study. First, follow-up was only collected until 24 weeks. Given the timing requirement to deliver the delayed intervention control group, a longer follow-up was not possible. Another limitation was limited racial diversity of the sample. Although rural communities are becoming more diverse, these communities in upstate New York were still predominantly white. Program results in the rural South and Southwest may differ. While about two-thirds of participants in the intervention arm participated in 50% or more of the classes, about a third did not. A better understanding of lack of adherence among these participants may help improve the intervention.

In addition, the missing data rate post-intervention was higher than expected. This was likely due to a move towards centralized study management for cost efficiencies of SHHC-2.0 for data collection reminders and scheduling. While attrition was underestimated, selection bias was not apparent in this study. The “tipping point” sensitivity analysis found that those lost to follow-up in the intervention group would have to decrease their average MVPA by 28 min per week more than the control group during the 24-week intervention period to reverse the findings.

## Conclusions

The findings in this report demonstrate that SHHC-2.0 was successful in increasing physical activity among previously sedentary, at-risk older, rural women at both 12 and 24 weeks. Future iterations may test a consolidated (e.g., 12-week) and/or virtually delivered version of the program to maximize impact and minimize cost and burden on participants. Finally, an important strength of SHHC-2.0 has been its commitment to feasibility – the program is designed to be conducted in a variety of community spaces with modest needs for equipment allowing it to be easily disseminated across a variety of settings. Future research should focus on testing this intervention in more diverse populations across the United States. The feasibility of dissemination of this intervention throughout state Extension networks should be explored.

## Supplementary Information

**Additional file 1:** **Table S1.** Comparison of models (Multiple Imputation, Complete Case, and Outliers removed) of within-group change and between-group change in physical activity behaviors and psychosocial measures from baseline to midpoint (12 weeks). **Table S2.** Comparison of models (Multiple Imputation, Complete Case, and Outliers removed) of within-group change and between-group change in physical activity behaviors and psychosocial measures from baseline to intervention end point (24 weeks). **Table S3.** Subsample of 60 years and older participants: within-group change and between-group change in physical activity behaviors and psychosocial measures from baseline to midpoint (12 weeks) and intervention end point (24 weeks).

## Data Availability

Data are available by request from the corresponding author.

## Abbreviations

BMI: Body mass index
FMI: Fraction of missing information
IPAQ-SF: International Physical Activity Questionnaire-Short Form
MET: Metabolic equivalent of task
MI: Multiple imputation
MVPA: Moderate to vigorous intensity physical activity
RUCA: Rural–Urban Commuting Area
SBQ: Sedentary Behavior Questionnaire
SHHC: Strong Hearts, Healthy Communities

## References

[1] Warburton DER, Bredin SSD (2017). Health benefits of physical activity: a systematic review of current systematic reviews. Curr Opin Cardiol.

[2] Sun F, Norman IJ, While AE. Physical activity in older people: a systematic review. BMC Public Health. 2013;13:1–17. 10.1186/1471-2458-13-449 PMC3651278 23648225

[3] Piercy KL, Troiano RP, Ballard RM, Carlson SA, Fulton JE, Galuska DA (2018). The physical activity guidelines for Americans. JAMA.

[4] National Center for Health Statistics. National Health Interview Survey, Sample Adult Core Component. 2018.

[5] Whitfield GP, Carlson SA, Ussery EN, Fulton JE, Galuska DA, Petersen R (2019). Trends in meeting physical activity guidelines among urban and rural dwelling adults - United States, 2008–2017. MMWR Morb Mortal Wkly Rep.

[6] Leider JP, Meit M, Mac McCullough J, Resnick B, Dekker D, Alfonso YN (2020). The state of rural public health: enduring needs in a new decade. Am J Public Health.

[7] Abildso CG, Daily SM, Meyer MRU, Edwards MB, Jacobs L, McClendon M (2021). Environmental factors associated with physical activity in rural US counties. Int J Environ Res Public Health.

[8] Sriram U, Morgan EH, Graham ML, Folta SC, Seguin RA (2018). Support and sabotage: a qualitative study of social influences on health behaviors among rural adults. J Rural Health.

[9] Folta SC, Goldberg JP, Lichtenstein AH, Seguin R, Reed PN, Nelson ME (2008). Factors related to cardiovascular disease risk reduction in midlife and older women: a qualitative study. Prev Chron Dis.

[10] Vasudevan A, Ford E (2021). Motivational factors and barriers towards initiating and maintaining strength training in women: a systematic review and meta-synthesis. Prev Sci.

[11] Olsen J (2013). An integrative review of literature on the determinants of physical activity among rural women. Public Health Nurs.

[12] Zimmermann K, Carnahan LR, Peacock NR (2016). Age-associated perceptions of physical activity facilitators and barriers among women in rural southernmost Illinois. Prev Chron Dis.

[13] Lo BK, Morgan EH, Folta SC, Graham ML, Paul LC, Nelson ME (2017). Environmental influences on physical activity among rural adults in Montana, United States: views from built environment audits, resident focus groups, and key informant interviews. Int J Environ Res Public Health.

[14] Krummel DA, Humphries D, Tessaro I (2002). Focus groups on cardiovascular health in rural women: implications for practice. J Nutr Educ Behav.

[15] Tessaro I, Rye S, Parker L, Trangsrud K, Mangone C, McCrone S (2006). Cookin’Up Health: developing a nutrition intervention for a rural Appalachian population. Health Promot Pract.

[16] Seguin R, Connor L, Nelson M, LaCroix A, Eldridge G (2014). Understanding barriers and facilitators to healthy eating and active living in rural communities. J Nut Metab.

[17] National Cancer Institute. Physical Activity Evidence-Based Program Listing. https://ebccp.cancercontrol.cancer.gov/topicPrograms.do?topicId=102268&choice=default. Accessed 6 Sept 2022.

[18] Seguin RA, Eldridge G, Graham ML, Folta SC, Nelson ME, Strogatz D (2016). Strong Hearts, Healthy Communities: a rural community-based cardiovascular disease prevention program. BMC Public Health.

[19] Folta SC, Paul L, Nelson ME, Strogatz D, Graham M, Eldridge GD (2019). Changes in diet and physical activity resulting from the Strong Hearts, Healthy Communities randomized cardiovascular disease risk reduction multilevel intervention trial. Int J Behav Nutr Phys Act.

[20] Pullyblank K, Strogatz D, Folta SC, Paul L, Nelson ME, Graham M (2020). Effects of the Strong Hearts, Healthy Communities Intervention on functional fitness of rural women. J Rural Health.

[21] Sriram U, Sandreuter K, Graham M, Folta S, Pullyblank K, Paul L (2019). Process evaluation of Strong Hearts, Healthy Communities: a rural community-based cardiovascular disease prevention program. J Nutr Educ Behav.

[22] United States Department of Agriculture Economic Research Service. 2010 Rural-Urban Commuting Area (RUCA) Codes. https://www.ers.usda.gov/data-products/rural-urban-commuting-area-codes/documentation/. Accessed 6 Sept 2022.

[23] Seguin RA, Graham ML, Eldridge G, Nelson ME, Strogatz D, Folta SC (2019). Strong Hearts for New York: a multilevel community-based randomized cardiovascular disease risk reduction intervention for rural women. Contemp Clin Trials.

[24] Seguin-Fowler RA, Eldridge GD, Rethorst C, Graham M, Demment M, Strogatz D (2022). Improvements and maintenance of clinical and functional measures among rural women: Strong Hearts, Healthy Communities-2.0 cluster randomized trial. Circ Cardiovasc Qual Outcomes..

[25] Seguin RA, Paul L, Folta SC, Nelson ME, Strogatz D, Graham ML (2018). Strong Hearts, Healthy Communities: a community-based randomized trial for rural women. Obesity.

[26] U.S. Department of Health and Human Services, Physical Activity Guidelines for Americans. Washington, DC: U.S. Department of Health and Human Services; 2018. https://health.gov/sites/default/files/2019-09/Physical_Activity_Guidelines_2nd_edition.pdf. Accessed 6 Sept 2022.

[27] Brooke P, Bullock R (1999). Validation of a 6 item cognitive impairment test with a view to primary care usage. Int J Geriatr Psychiatry.

[28] Choi L, Ward SC, Schnelle JF, Buchowski MS (2012). Assessment of wear/nonwear time classification algorithms for triaxial accelerometer. Med Sci Sports Exerc.

[29] Freedson PS, Melanson E, Sirard J (1998). Calibration of the computer science and applications. Inc accelerometer Med Sci Sports Exerc.

[30] International Physical Activity Questionnaire. Guidelines for Data Processing and Analysis of the International Physical Activity Questionnaire. http://www.ipaq.ki.se. Accessed 6 Sept 2022.

[31] Rosenberg DE, Norman GJ, Wagner N, Patrick K, Calfas KJ, Sallis JF (2010). Reliability and validity of the Sedentary Behavior Questionnaire (SBQ) for adults. J Phys Act Health.

[32] Sallis JF, Pinski RB, Grossman RM, Patterson TL, Nader PR (1988). The development of self-efficacy scales for health-related diet and exercise behaviors. Health Educ Res.

[33] Sallis JF, Grossman RM, Pinski RB, Patterson TL, Nader PR (1987). The development of scales to measure social support for diet and exercise behaviors. Prev Med.

[34] Roper A (2002). Exercise attitudes and behaviors: a survey of adults age 50–79.

[35] Yuan Y (2010). Multiple Imputation for Missing Data: Concepts and New Developments (Version 9.0).

[36] Kohl HW (2001). Physical activity and cardiovascular disease: evidence for a dose response. Med Sci Sports Exerc.

[37] Aune D, Norat T, Leitzmann M, Tonstad S, Vatten LJ (2015). Physical activity and the risk of type 2 diabetes: a systematic review and dose-response meta-analysis. Eur J Epidemiol.

[38] Dunn AL, Trivdei MH, O'Neal HA (2001). Physical activity dose-response effects on outcomes of depression and anxiety. Med Sci Sports Exerc.

[39] Lee IM, Skerrett PJ (2001). Physical activity and all-cause mortality: what is the dose-response relation?. Med Sci Sports Exerc.

[40] Cunningham SA, Vaquera E, Maturo CC, Narayan KMV (2012). Is there evidence that friends influence body weight? A systematic review of empirical research. Soc Sci Med.

[41] Gorin AA, Wing RR, Fava JL, Jakicic JM, Jeffery R, West DS (2008). Weight loss treatment influences untreated spouses and the home environment: evidence of a ripple effect. Int J Obesity.

[42] Rossini R, Moscatiello S, Tarrini G, Di Domizio S, Soverini V, Romano A (2011). Effects of cognitive-behavioral treatment for weight loss in family members. J Am Diet Assoc.

[43] Nigg CR, Borrelli B, Maddock J, Dishman RK (2008). A theory of physical activity maintenance. Appl Psychol.

