# Supplementary material for: Changes in physical activity outcomes in the Strong Hearts, Healthy Communities (SHHC-2.0) community-based randomized trial
Source: Int J Behav Nutr Phys Act. 2022 Dec 28;19:159. doi: 10.1186/s12966-022-01401-1 (PMC9795747; doi:10.1186/s12966-022-01401-1)
Supplement: Supplementary file 1 — Additional file 1: Table S1. Comparison of models (Multiple Imputation, Complete Case, and Outliers removed) of within-group change and between-group change in physical activity behaviors and psychosocial measures from baseline to midpoint (12 weeks). Table S2. Comparison of models (Multiple Imputation, Complete Case, and Outliers removed) of within-group change and between-group change in physical activity behaviors and psychosocial measures from baseline to intervention end point (24 weeks). Table S3. Subsample of 60 years and older participants: within-group change and between-group change in physical activity behaviors and psychosocial measures from baseline to midpoint (12 weeks) and intervention end point (24 weeks). [file 12966_2022_1401_MOESM1_ESM.docx]

**SUPPLEMENTAL**

**Table S1. Comparison of Models (Multiple Imputation, Complete Case, and Outliers removed) of Within-Group Change and Between-Group Change in Physical Activity Behaviors and Psychosocial Measures from Baseline to Midpoint (12 weeks)**

|  | **Multiple imputation**  **(presented in paper)**  **(n=182)** | | **Complete Case** | | **Outlier Analysis** | |
| --- | --- | --- | --- | --- | --- | --- |
|  | Estimate (95%CI) | *P* value | Estimate (95%CI) | *P* value | Estimate (95%CI) | *P* value |
| **Physical Activity Behaviors (Accelerometry)** |  |  |  |  |  |  |
| Average Light Physical Activity (minutes/day) ***** | **25.67 (2.36,48.97)** | **0.031** | 17.43 (-4.43,39.29) | 0.117 | **25.18 (2.37,47.99)** | **0.031** |
| Average Moderate to Vigorous Physical Activity (minutes/day) ‖ | **8.13 (3.87,12.40)** | **<0.001** | **8.01 (3.74,12.28)** | **<0.001** | **8.12 (4.20,12.03)** | **<0.001** |
| **Physical Activity Behaviors (Survey)** |  |  |  |  |  |  |
| IPAQ total MET minutes per week † | **725.77 (242.96,1208.59)** | **0.003** | **768.06 (213.83,1322.29)** | **0.007** | **775.48 (294.54,1256.41)** | **0.002** |
| IPAQ walking MET minutes per week † | 81.77 (-133.59,297.13) | 0.457 | 72.04 (-154.71,298.79) | 0.531 | 80.90 (-140.14,301.94) | 0.473 |
| IPAQ moderate MET minutes per week † | **280.91 (103.36,458.46)** | **0.002** | **367.07 (212.94,521.20)** | **<0.001** | **312.92 (158.43,467.40)** | **<0.001** |
| IPAQ vigorous MET minutes per week † | **363.09 (45.96,680.21)** | **0.025** | **380.2 (40.01,720.38)** | **0.029** | **366.89 (42.08,691.71)** | **0.027** |
| SBQ: Total sitting hours per week † | -3.20 (-11.22,4.83) | 0.434 | -5.09 (-12.68,2.50) | 0.187 | -3.10 (-11.15,4.96) | 0.451 |
| **Physical activity psychosocial measures (Survey)** |  |  |  |  |  |  |
| Self-efficacy for sticking to exercise habits (scale 1-5) † | **0.54 (0.22,0.87)** | **0.001** | **0.51 (0.19,0.82)** | **0.002** | **0.52 (0.19,0.84)** | **0.002** |
| Self-efficacy for making time for exercise (scale 1-5) † | 0.28 (-0.05,0.61) | 0.091 | 0.24 (-0.08,0.56) | 0.146 | 0.23 (-0.08,0.55) | 0.148 |
| Family participation in exercise (scale 10 to 50) ‡ | **3.87 (0.53,7.20)** | **0.023** | **4.18 (1.39,6.97)** | **0.004** | **3.78 (0.90,6.67)** | **0.010** |
| Friend participation in exercise (scale 10 to 50) § | **7.18 (3.00,11.36)** | **0.001** | **6.50 (2.50,10.51)** | **0.002** | **7.08 (3.16,11.00)** | **<0.001** |
| Family rewards and punishment for exercise (scale 3 to 15) ‡ | -0.11 (-1.14,0.92) | 0.838 | -0.17 (-0.65,0.31) | 0.493 | -0.03 (-0.53,0.48) | 0.922 |
| Combined attitude toward exercise score (scale 1-14) † | -0.11 (-0.24,0.02) | 0.094 | **-0.15 (-0.27,-0.03)** | **0.019** | -0.11 (-0.23,0.02) | 0.104 |

All estimates adjusted for random cluster (community) effects, random assignment group, age, and education.

**Bold** indicates P value<0.05

*CI* Confidence Interval, *IPAQ* International Physical Activity Questionnaire, *MET* Metabolic Equivalent of Task, *SBQ* Sedentary Behavior Questionnaire

***** Data available for 125 of 182 total for complete case models.

‖ Data available for 132 of 182 total for complete case models.

† Data available for 130 of 182 total for complete case models.

‡ Data available for 110 of 182 total for complete case models.

§ Data available for 104 of 182 total for complete case models.

**Table S2. Comparison of Models (Multiple Imputation, Complete Case, and Outliers removed) of Within-Group Change and Between-Group Change in Physical Activity Behaviors and Psychosocial Measures from Baseline to Intervention End Point (24 weeks)**

|  | **Multiple imputation (presented in paper)**  **(n=182)** | | **Complete Case** | | **Outlier Analysis** | |
| --- | --- | --- | --- | --- | --- | --- |
|  | Estimate (95%CI) | *P* value | Estimate (95%CI) | *P* value | Estimate (95%CI) | *P* value |
| **Physical Activity Behaviors (Accelerometry)** |  |  |  |  |  |  |
| Average Light Physical Activity (minutes/day) ***** | 1.21 (-21.80,24.21) | 0.918 | -4.30 (-25.36,16.77) | 0.690 | 1.83 (-20.28,23.95) | 0.871 |
| Average Moderate to Vigorous Physical Activity (minutes/day) ***** | **6.43 (1.48,11.38)** | **0.011** | **6.57 (1.67,11.47)** | **0.010** | **6.35 (1.96,10.75)** | **0.005** |
| **Physical Activity Behaviors (Survey)** |  |  |  |  |  |  |
| IPAQ total MET minutes per week † | **955.90 (355.93,1555.86)** | **0.002** | **936.14 (227.50,1644.77)** | **0.011** | **962.76 (353.76,1571.76)** | **0.002** |
| IPAQ walking MET minutes per week † | 191.50 (-47.31,430.31) | 0.116 | 239.40 (-39.43,518.22) | 0.095 | 202.86 (-32.70,438.42) | 0.091 |
| IPAQ moderate MET minutes per week † | **269.82 (27.01,512.63)** | **0.029** | 241.19 (-62.07,544.45) | 0.122 | **271.25 (20.33,522.18)** | **0.034** |
| IPAQ vigorous MET minutes per week † | **494.58 (158.12,831.04)** | **0.004** | **455.55 (64.60,846.50)** | **0.024** | **498.95 (162.01,835.90)** | **0.004** |
| SBQ: Total sitting hours per week † | 1.67 (-7.58,10.93) | 0.723 | 1.06 (-7.80,9.93) | 0.814 | 1.61 (-7.11,10.34) | 0.717 |
| **Physical activity psychosocial measures (Survey)** |  |  |  |  |  |  |
| Self-efficacy for sticking to exercise habits (scale 1-5) † | 0.33 (-0.09,0.75) | 0.124 | 0.18 (-0.18,0.55) | 0.332 | 0.32 (-0.05,0.70) | 0.092 |
| Self-efficacy for making time for exercise (scale 1-5) † | 0.29 (-0.13,0.72) | 0.174 | 0.14 (-0.25,0.52) | 0.488 | 0.28 (-0.11,0.67) | 0.161 |
| Family participation in exercise (scale 10 to 50) ‡ | **5.69 (1.78,9.61)** | **0.005** | **5.71 (2.60,8.82)** | **0.001** | **5.65 (2.15,9.15)** | **0.002** |
| Friend participation in exercise (scale 10 to 50) § | 4.46 (-0.08,9.01) | 0.054 | 3.58 (-0.16,7.33) | 0.064 | **4.39 (0.01,8.77)** | **0.049** |
| Family rewards and punishment for exercise (scale 3 to 15) ‡ | -0.06 (-2.18,2.06) | 0.954 | 0.10 (-0.52,0.72) | 0.758 | 0.10 (-0.60,0.81) | 0.775 |
| Combined attitude toward exercise score (scale 1-14) † | **-0.18 (-0.34,-0.01)** | **0.033** | **-0.20 (-0.34,-0.06)** | **0.005** | **-0.17 (-0.32,-0.03)** | **0.020** |

All estimates adjusted for random cluster (community) effects, random assignment group, age, and education.

**Bold** indicates P value<0.05

*CI* Confidence Interval, *IPAQ* International Physical Activity Questionnaire, *MET* Metabolic equivalent task, *SBQ* Sedentary Behavior Questionnaire

***** Data available for 119 of 182 total for complete case models.

† Data available for 114 of 182 total for complete case models.

‡ Data available for 95 of 182 total for complete case models.

§ Data available for 91 of 182 total for complete case models.

**Table S3. Subsample of 60 years and older Participants: Within-Group Change and Between-Group Change in Physical Activity Behaviors and Psychosocial Measures from Baseline to Midpoint (12 weeks) and Intervention End Point (24 weeks)**

|  | **0 to 12 weeks** | | | | **0 to 24 weeks** | | | |
| --- | --- | --- | --- | --- | --- | --- | --- | --- |
|  | Control participants | Intervention participants | Difference between groups | | Control participants | Intervention participants | Difference between groups | |
|  | Mean change ± SD | Mean change ±SD | Estimate (95%CI) | *P* value | Mean change ± SD | Mean change ±  SD | Estimate (95%CI) | *P* value |
| **Physical Activity Behaviors (Accelerometry)** |  |  |  |  |  |  |  |  |
| Average Light Physical Activity (minutes/day) | 2.7 ± 12.1 | 19.4 ± 12.6 | 16.77 (-19.74,53.28) | 0.368 | 9.4 ± 12.9 | 17.7 ± 12.9 | 5.72 (-30.81,42.24) | 0.759 |
| Average Moderate to Vigorous Physical Activity (minutes/day) | -0.8 ± 2.2 | 7.3 ± 2.0 | **8.15 (2.17,14.13)** | **0.008** | -0.9 ± 2.4 | 5.7 ± 1.8 | **7.00 (0.65,13.36)** | **0.031** |
| **Physical Activity Behaviors (Survey)** |  |  |  |  |  |  |  |  |
| IPAQ total MET minutes per week | 272.9 ± 212.5 | 790.3 ± 384.6 | 517.36 (-369.89,1404.62) | 0.253 | 103.8 ± 214.5 | 896.1 ± 411.8 | 794.07 (-223.02,1811.15) | 0.126 |
| IPAQ walking MET minutes per week | 138.1 ± 106.4 | 149.8 ± 143.2 | 11.63 (-347.21,370.48) | 0.949 | 70.9 ± 124.8 | 260.9 ± 160.0 | 198.39 (-234.95,631.73) | 0.369 |
| IPAQ moderate MET minutes per week | -23.8 ± 78.8 | 216.2 ± 163.6 | 240.03 (-129.29,609.36) | 0.203 | -24.7 ± 99.7 | 216.1 ± 194.1 | 241.01 (-215.38,697.4) | 0.301 |
| IPAQ vigorous MET minutes per week | 158.6 ± 147.1 | 424.3 ± 195.8 | 265.70 (-236.29,767.68) | 0.299 | 57.6 ± 113.4 | 419.2 ± 220.7 | 354.66 (-163.19,872.52) | 0.179 |
| SBQ: Total sitting hours per week | -8.8 ± 4.7 | -10.8 ± 4.1 | -2.02 (-15.22,11.17) | 0.764 | -13.1 ± 5.0 | -8.4 ± 5.5 | 4.42 (-11.14,19.99) | 0.577 |
| **Physical activity psychosocial measures (Survey)** |  |  |  |  |  |  |  |  |
| Self-efficacy for sticking to exercise habits (scale 1-5) | -0.5 ± 0.2 | 0.3 ± 0.2 | **0.83 (0.31,1.36)** | **0.002** | -0.5 ± 0.2 | 0.1 ± 0.2 | 0.54 (-0.11,1.19) | 0.104 |
| Self-efficacy for making time for exercise (scale 1-5) | -0.5 ± 0.2 | -0.1 ± 0.1 | 0.47 (-0.05,0.99) | 0.076 | -0.6 ± 0.3 | 0.04 ± 0.2 | 0.54 (-0.14,1.23) | 0.121 |
| Family participation in exercise (scale 10 to 50) | 0.1 ± 1.6 | 1.7 ± 1.4 | 1.56 (-3.19,6.30) | 0.519 | 0.6 ± 1.9 | 4.7 ± 2.5 | 3.90 (-2.41,10.21) | 0.225 |
| Friend participation in exercise (scale 10 to 50) | -2.0 ± 2.3 | 4.6 ± 2.4 | 6.61 (-0.37,13.59) | 0.064 | 0.5 ± 2.6 | 4.0 ± 2.0 | 3.61 (-3.15,10.36) | 0.294 |
| Family rewards and punishment for exercise (scale 3 to 15) | 0.1 ± 0.9 | -0.3 ± 0.5 | -0.31 (-2.46,1.85) | 0.780 | 0.2 ± 0.6 | 0.3 ± 0.7 | -0.13 (-3.26,3.01) | 0.937 |
| Combined attitude toward exercise score (scale 1-14) | -0.1 ± 0.1 | -0.1 ± 0.1 | -0.07 (-0.29,0.16) | 0.573 | -0.1 ± 0.1 | -0.3 ± 0.1 | -0.15 (-0.41,0.10) | 0.242 |

All estimates from MI models and adjusted for random cluster (community) effects, random assignment group, age, and education.

**Bold** indicates P value<0.05

*CI* Confidence Interval*, IPAQ* International Physical Activity Questionnaire, *MET* Metabolic Equivalent of Task, *MI* Multiple Imputation, *SBQ* Sedentary Behavior Questionnaire, *SD* Standard Deviation
